# Quetiapine-Induced Mania and Hypomania: A Case Study and Literature Review

**DOI:** 10.1192/j.eurpsy.2025.1075

**Published:** 2025-08-26

**Authors:** S. Prasad, S. Das, S. Gunturu

**Affiliations:** 1 Psychiatry, BronxCare Health System, New York, United States; 2 Psychiatry, Western Health, Melbourne, Australia

## Abstract

**Introduction:**

In the realm of mental health treatment, quetiapine, is a commonly prescribed for various conditions like schizophrenia, acute manic episodes, anxiety disorder, and major depressive disorder. Its pharmacological profile includes serotonin, dopamine, histamine, and adrenergic receptor antagonism, with a higher affinity for 5-HT2A receptors compared to D2 receptors. Quetiapine’s unique receptor interactions and low risk of side effects make it a versatile option for clinicians.

**Objectives:**

This case report aims to shed light on the potential for quetiapine, an atypical antipsychotic, to induce or exacerbate manic or hypomanic symptoms in patients with bipolar affective disorder. By presenting a real-world case study and reviewing existing literature, we seek to contribute to the understanding of how quetiapine dosage can influence mood symptomatology and inform clinical decision-making regarding treatment strategies in bipolar disorder.

**Methods:**

This investigation was conducted following SANRA guidelines for case reports, and involved performing a search in PubMed to compile evidence on how quetiapine impacts mood symptoms, particularly at different dosage levels, in individuals with bipolar affective disorder.

**Results:**

In our case involving a 26-year-old male with bipolar affective disorder, he experienced a manic episode when starting quetiapine at 200 mg/day, with symptom resolution after discontinuation of the medication. Patients ranged from 21 to 66 years old, with males being more prevalent (11 out of 15 cases). The most common diagnoses were paranoid schizophrenia (8 cases) and bipolar disorder (4 cases), with two bipolar patients experiencing worsened hypomanic symptoms. Dosages of quetiapine varied from 100 mg/day to 600 mg/day, with symptoms appearing between the 2nd day and 7th week of starting treatment. Most cases (13 out of 15) were managed by stopping quetiapine, while one patient’s dose was increased to 800 mg/day and another reduced to 100 mg/day. Discontinuing quetiapine in the studied patient led to symptom improvement within two weeks, aligning with similar outcomes in other cases.Alternative antipsychotics were effective if quetiapine worsened symptoms. Various theories explain quetiapine’s impact, with poor metabolizers potentially needing higher doses for relief.

**Conclusions:**

Despite the risk of inducing hypomanic/manic symptoms at lower doses of quetiapine, findings suggest that in randomized placebo-controlled studies on bipolar patients treated for depressive episodes, quetiapine has shown a lower likelihood of causing a manic or hypomanic switch compared to a placebo. Clinicians should cautiously monitor patients during the initial dose titration period but can consider quetiapine as a management option for bipolar disorder.

**Disclosure of Interest:**

None Declared

